# The Effect of Creatine Supplementation on Lean Body Mass with and Without Resistance Training

**DOI:** 10.3390/nu17061081

**Published:** 2025-03-19

**Authors:** Imtiaz Desai, Anurag Pandit, Abbie E. Smith-Ryan, David Simar, Darren G. Candow, Nadeem O. Kaakoush, Amanda D. Hagstrom

**Affiliations:** 1 School of Health Sciences, Faculty of Medicine and Health, University of New South Wales, Sydney, NSW 2052, Australia; 2 Centre for Pain IMPACT, Neuroscience Research Australia, Sydney, NSW 2031, Australia; 3 Department of Exercise and Sport Science, University of North Carolina at Chapel Hill, Chapel Hill, NC 27599, USA; 4 Faculty of Kinesiology and Health Studies, University of Regina, Regina, SK S4S 0A2, Canada; 5 School of Biomedical Sciences, University of New South Wales, Sydney, NSW 2052, Australia

**Keywords:** nutrition, strength training, hypertrophy

## Abstract

**Background/Objectives**: Creatine monohydrate (CrM) is considered to be one of the most effective supplements for enhancing lean body mass during resistance training. However, CrM may influence body water content, potentially confounding lean body mass measurements. Therefore, this randomised controlled trial assessed the effect of CrM alone on lean body mass following a supplement wash-in, and when combined with a resistance training program. **Methods**: Sixty-three (34 females, 29 males, 31 ± 8 years) participants were randomised to supplement with CrM (5 g/day for 13 weeks: wash-in + 12-week resistance training) or serve as a control (received no creatine or placebo). Lean body mass was measured using dual X-ray absorptiometry at baseline, post 7-day wash-in, and post 12 weeks of resistance training. Both groups began the same training program post CrM wash-in. **Results**: After the 7-day wash-in, the supplement group gained 0.51 ± 1.79 kg more lean body mass than the control group (*p* = 0.03). Following the wash-in, both groups gained 2 kg after resistance training (*p* < 0.0001), with no between-group difference in lean body mass growth (*p* = 0.71). Sex-disaggregated analysis showed that the supplement group, only in females, gained 0.59 ± 1.61 kg more lean body mass than the controls (*p* = 0.04). There were no group differences in lean body mass growth following resistance training in females (*p* = 0.10) or males (*p* = 0.35). **Conclusions**: A 7-day CrM wash-in increased lean body mass, particularly in females. Thereafter, CrM did not enhance lean body mass growth when combined with resistance training, likely due to its short-term effects on lean body mass measurements. A maintenance dose of higher than 5 g/day may be necessary to augment lean body mass growth.

## 1. Introduction

The benefits of improving lean body mass (LBM) are far reaching, including reduced all-cause mortality [1], better bone health [2,3], and improved glycaemic control [4]. Resistance training (RT) is well established as the gold-standard method of stimulating LBM growth, with an average increase in LBM of ~1.5 kg expected following training periods of approximately three to four months [5,6]. There are an array of dietary methods proposed to augment RT adaptations [7], including long-term creatine monohydrate (CrM) supplementation, which has been shown to increase LBM by ~1.1 kg more than RT alone in healthy adults 18 to 47 years old [8,9]. The larger LBM growth with CrM has been proposed to be the result of greater training volume during RT, thereby eliciting a larger growth stimulus [10]. Although CrM may influence LBM growth by enhancing the growth factors, processes, and pathways involved in the translation of muscle proteins [11,12], there is currently no direct evidence that CrM increases the rates of protein synthesis [13]. Furthermore, the effects of CrM alone on LBM compared to RT have only been examined in older adults [14]. Therefore, there is no original research elucidating the relationship between long-term CrM supplementation and LBM gains in the absence of exercise in healthy individuals aged under 50 years.

**Figure 1:**
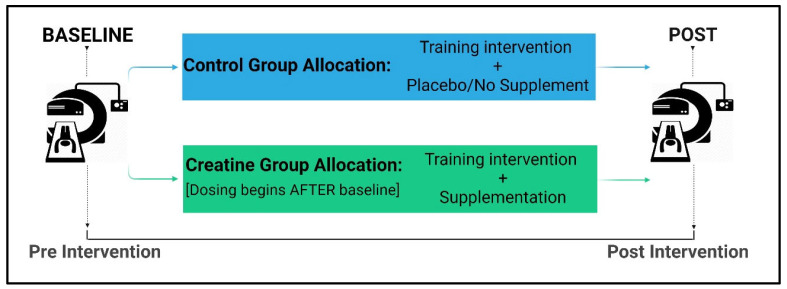
Previous study designs utilised in exercise science and nutrition research.

CrM dosing strategies include ‘loading’ and ‘maintenance’ phases. Loading involves supplementing with 20–25 g/day for five to seven days prior to a ‘maintenance’ dose of 2–5 g/day. Loading is used to rapidly saturate intramuscular creatine stores [15]; thus, it is believed to optimise the ergogenic benefits of supplementation. Consuming 2–5 g/day for at least four weeks has been established as sufficient for increasing intramuscular creatine stores [15] as well as muscle size [16,17]. Given that the rise in intramuscular creatine stores occur more slowly with maintenance doses, the approach may be more effective for increasing LBM growth with long-term RT [10,18]. However, studies reporting a significant relationship between 2–5 g/day and greater LBM growth included loading phases. Thus, it is unclear whether the slower rise in intramuscular creatine stores with long-term maintenance doses, without prior loading phases, do augment LBM growth with RT. There is some evidence to suggest that, since creatine uptake occurs via a sodium-dependant transporter (SLC6A8) [19], the rapid increase in muscle creatine from loading protocols may induce net (acute) water retention to compensate for changes in intracellular osmolality. However, this evidence remains questionable. Studies of 5–6 weeks in duration showed no increases in intracellular (ICW), extracellular (ECW), or total body water (TBW) [20,21,22,23]. Of these, all but one study [22] used a loading phase. In contrast, Powers et al. [24] and Ribeiro et al. [25], respectively, showed significant increases in TBW after 4 weeks, and all fluid compartments after 8 weeks of supplementation inclusive of loading phases. Importantly, these studies were all designed with baseline measurements conducted before commencing long-term supplementation (Figure 1), which did not account for potential changes to LBM measurements from CrM alone.

To date, only one study has investigated the short-term effects of supplementation in the absence of exercise. Safdar et al. [26] measured changes in TBW, fat-free mass (LBM plus bone minerals), mRNA expression, and protein content in skeletal muscle after 10 days of CrM supplementation alone (20 g/day for 3 days; 5 g/day for 7 days). Over time, CrM significantly increased TBW and fat-free mass. The authors proposed that differential gene expression with 10 days of CrM supplementation reflected a homeostatic adaptation to changes in cellular osmolarity. Based on these findings, it appears that short-term CrM supplementation has the potential to impact measurements of LBM, independent of training and/or dietary intervention. Therefore, it is possible that the larger increases in LBM found when CrM is combined with RT in adults [8] are partly due to the impact of short-term CrM alone on measurements of LBM and are not entirely indicative of gains in LBM.

The purpose of this study was therefore to investigate whether whole-body and segmental LBM measurements were affected by a seven-day wash-in of 5 g/day CrM in the absence of RT in healthy, untrained adults. It was hypothesised that the 7-day CrM wash-in phase would significantly increase LBM measurements. We then sought to investigate whether the wash-in LBM outcomes impacted changes in LBM following supplementation with a 12-week RT. We hypothesised that an increase in measured LBM after the wash-in would affect the magnitude of LBM growth after the ensuing CrM and RT intervention.

## 2. Materials and Methods

This study was included as part of a larger single-blind, controlled, randomised trial (Australia New Zealand Clinical Trial Registry number ACTRN12622000040763). The research protocol was approved by the University of New South Wales Human Research Ethics Committee on 2 December 2021 (approval number HC210725).

### 2.1. Experimental Approach

**Figure 2:**
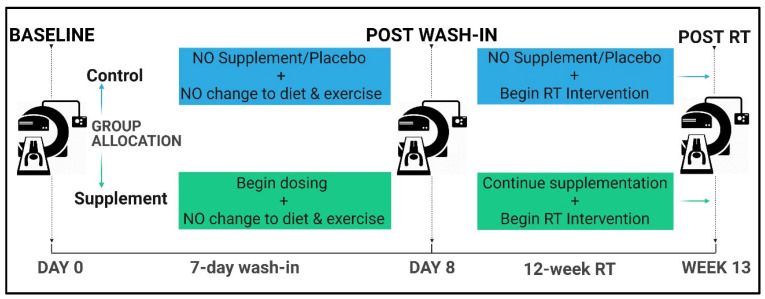
Current study design; RT = resistance training.

This randomised controlled trial was conducted over a period of 13 weeks. Assessments were conducted at 3 time points (T): (T1) baseline; (T2) post 7-day non-exercise wash-in phase; (T3) post 12-week RT intervention (Figure 2). This created 3 stages for the intervention: 7-day non-exercise wash-in (T1 to T2); 12-week RT intervention only (T2 to T3); wash-in and RT intervention (T1 to T3). For all assessments, participants reported to the Exercise Physiology Research Lab (UNSW Sydney, Australia) at the same time of day between 8 h and 9 h following an 8-h fast which included refraining from all liquids except water. Hydration status and amount of water consumed was not controlled or measured. Participants were additionally instructed to refrain from exercise for 12 h prior; to wear exercise clothing free from zips, buckles, or metal; to remove jewellery; and to void the bladder within 30 min prior to the assessment. Following the first assessment, participants were randomised into the supplementation or control group. Block randomisation for every ten participants was generated by using the website Randomization.com [27].

### 2.2. Participants

**Figure 3:**
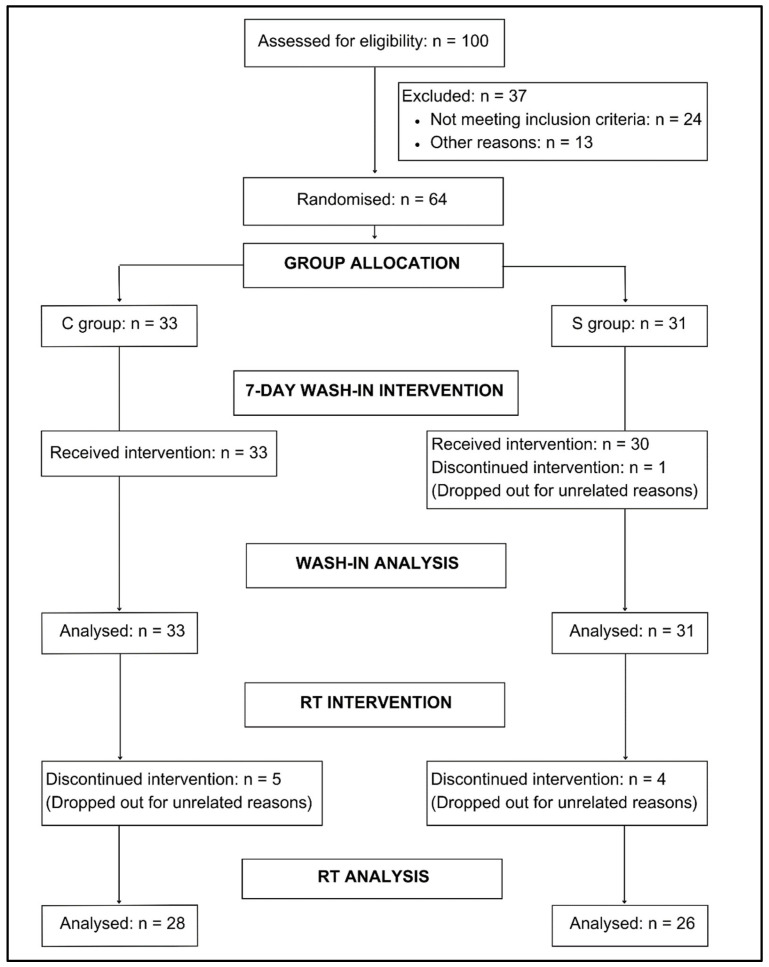
Study flowchart CONSORT diagram.

Individuals interested in participating were screened for eligibility with an online pre-screen form using the Research Electronic Data Capture (REDCap) tool hosted at The University of New South Wales [28,29]. The form included Stage 1 of the Australian Adult Pre-Exercise Screening System (APSS) [30] to identify those who may have been at a higher risk of an adverse event due to exercise, and the International Physical Activity Questionnaire Short Form (IPAQ-S) [31] to evaluate physical activity levels. After an explanation of all procedures, risks, and benefits was provided, each participant provided written informed consent before participating. Figure 3 shows the study enrolment process.

Apparently healthy individuals (18 to 50 years), with a body mass index of ≤30 kg/m^2^ and not meeting current physical activity guidelines of at least 150 min of moderate-intensity exercise were included. The age-predicted maximum heart rate equation used was (220–age), as this is the method used in the APSS [30]. Individuals who undertook RT within the previous 12 months were excluded. Other exclusion criteria were the use of antibiotics or creatine supplementation in the previous 8 weeks before the study started, pregnancy, and the presence of any injury, disease, or chronic illness. Participant-descriptive information is presented in Table 1.

### 2.3. Procedures

#### 2.3.1. Body Composition Measurement

Whole-body and segmental (arm, leg, and trunk) LBM (kg) and fat mass (FM, kg) were measured using dual energy x-ray absorptiometry (DXA) (iDXA Series, GE Healthcare Lunar, Madison, WI, USA). The DXA underwent a quality-assurance check every second day, and monthly calibration using phantoms and instructions provided by the manufacturer (enCore software, version 15). Participants were instructed to wear the same clothing for the pre and post scans. Height to the nearest 0.1 cm and weight to the nearest 0.1 kg were first measured manually using a stadiometer and calibrated electronic scale (Seca, Birmingham, UK). Participants were asked to lay supine in anatomical position and were given verbal and tactile cues to align body positions. They were instructed to refrain from moving or talking until the scan was complete. The intraclass correlation coefficient (ICC) for test–retest reliability of DXA LBM measurements for this sample, assessed using a one-way random effects model, was 0.996, and the SEM was 0.59 kg.

#### 2.3.2. Diet and Physical Activity Logs

Participants completed a 3-day food log for the 3 days prior to each assessment (baseline, post seven-day wash-in phase, and post 12-week RT intervention) to provide an estimate of their typical total kilocalorie (kcal/day) and macronutrient (g/day of carbohydrate, fat, and protein) intake. Dietary logs were self-recorded by participants and shared with research personnel using the MyFitnessPal application (Under Armour Inc., Baltimore, MA, USA), which contains a large, detailed food database. The IPAQ-S [31] was used to assess changes in habitual physical activity across time. Participants were instructed to maintain their current dietary habits and physical activity levels for the duration of the study.

#### 2.3.3. Supplementation Protocol

Participants began supplementing with 5 g of pure, unsweetened CrM (True Protein, Brookvale, NSW 2100, Australia) after the first assessment. They were instructed to ingest the CrM once a day, dissolved in water at any normal mealtime. The amount of water consumed was not controlled for. Participants self-administered the CrM, and their compliance was collected weekly using REDCap (version 14.5.3) electronic data-capture tools [28,29]. All participants were instructed to not exercise during the wash-in period, and this was assessed by the IPAQ-S.

#### 2.3.4. Resistance Training Program

After the 7-day wash-in, both groups followed the same RT program that comprising 3 full-body sessions a week for 12 weeks (Supplementary Materials, Table S1). All sessions were supervised by tertiary qualified exercise physiologists and commenced with a standardised warm-up of dynamic flexibility exercises. Each session consisted of 5 exercises: 2 compound movements each for the upper and lower body, and 1 isolation movement for the upper body. Four sets were prescribed for all movements to ensure an adequate weekly training volume for hypertrophy [32]. Training intensities were 6 to 12 repetition maximums (RM) with 60 and 120 s of rest between sets and exercises, respectively. To adhere to the prescribed RM, an individual’s rating of perceived exertion (RPE) on a Likert scale of 1–10 was recorded. RPE corresponds to the number of repetitions an individual perceives they will be able to perform after the set is complete, where an RPE 5 equates to 5 reps more, RPE 6 is 4 reps more, RPE 7 is 3 reps more, and so on [33]. When a RPE of 8 or lower was recorded, the external load (kg) was adjusted on successive sets to ensure that subjects achieved the target RM. The RM method was used to ensure that training intensities were relative to the individual’s abilities while also standardising the training intensity across all participants [34].

#### 2.3.5. Statistical Analyses

Normality and equal variances were assessed using the Shapiro–Wilk and Levene’s tests, respectively. Independent *t*-tests were performed to determine whether baseline anthropometric and physical activity values were different between groups. Differences in whole-body and segmental LBM change between groups were analysed separately across the 3 stages of the intervention: 7-day non-exercise wash-in (T1 to T2); 12-week RT intervention only (T2 to T3); wash-in and RT intervention (T1 to T3). A linear regression model was used to analyse the differences in LBM changes between groups for each stage, with absolute LBM from the beginning of the respective stage used as a covariate to adjust for baseline differences. A linear mixed model (LMM) was used to assess differences in caloric and macronutrient consumption across groups and time points. The Wilcoxon rank–sum test and generalised linear model (GLM) were used instead of the independent *t*-test and the linear regression model, respectively, when data were not normal or variances were unequal. Effect size was demonstrated with partial eta squared (η_p_^2^) and interpreted as small (0.01–0.59), medium (0.06–0.139), or large (>0.14) [35]. A post hoc analysis was conducted to assess changes in all LBM outcomes with sexes stratified. This study was part of a larger randomised controlled trial assessing the effect of the gut microbiota on LBM growth following RT. Given that the effect size of the gut microbiota on LBM changes was unknown, an a priori power analysis was not conducted to calculate sample size. The intended sample size of 33 per group in the present study included a potential attrition rate of 10% and was based on sample sizes in similar studies [36,37,38].

Significance was set at *p* ≤ 0.05 and 95% confidence intervals were used. All analyses were conducted on R Studio version 4.3.2 [39] using the base stats, and emmeans (1.10.0) and SimplyAgree (0.1.2) packages. All data are presented as mean ± standard deviation (SD).

## 3. Results

One male from the CrM group withdrew after the baseline assessment for reasons unrelated to the study and was not included in the analysis. Five people from the control group (three females and two males) and four from the CrM group (two females and one male) withdrew after the second assessment for reasons unrelated to the study. No adverse events were reported during the intervention. Adherence to the CrM supplementation was 95%. Session attendance was 91.92% and exercise compliance was 99.91%. Self-reported carbohydrate consumption was greater in the control group at baseline, while there were no other differences in caloric or macronutrient intake between groups at each timepoint (Supplementary Materials, Table S2).

### 3.1. Body Composition

#### 3.1.1. Seven-Day Wash-In Phase (T1–T2)

**Figure 4:**
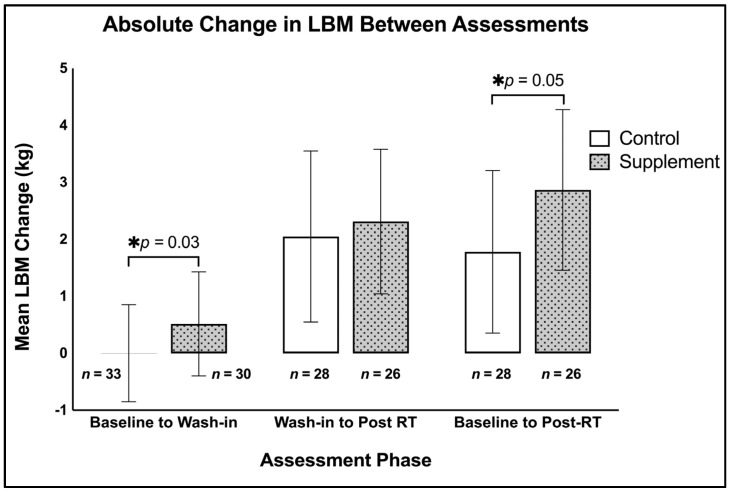
Absolute change (mean ± SD) in lean body mass (kg), LBM = lean body mass, RT = resistance training; * denotes *p* < 0.05.

After the CrM wash-in period without exercise, there was a significant difference between groups for changes in total LBM favouring the supplement group (*p* = 0.03; Supplement: ∆ 0.51 ± 1.26 kg; Control: ∆ 0 ± 1.20 kg; Figure 4). There were no between-group differences in the segmental arm (*p* = 0.26) or leg (*p* = 0.72) LBM results. There was a significant difference between groups for changes in trunk LBM favouring the supplement group (*p* = 0.01; supplement: ∆ 0.32 ± 0.92 kg; control: ∆ −0.10 ± 0.88 kg; see Supplementary Materials, Table S3). There were no between-group differences in fat mass (*p* = 0.27; Supplementary Materials, Table S4).

#### 3.1.2. Twelve-Week RT Intervention (T2–T3)

Following the wash-in, both groups gained 2 kg of total LBM after RT (*p* < 0.0001), whereas there were no differences in total LBM change between groups (*p* = 0.71; supplement: ∆ 2.24 ± 1.79 kg; control: ∆ 2.11 ± 1.71 kg; see Figure 4). Changes in segmental LBM were no different between groups (arm, *p* = 0.54; leg *p* = 0.54; trunk, *p* = 0.92; see Supplementary Materials, Table S3). There were no between-group differences in fat mass changes (*p* = 0.65; Supplementary Materials, Table S4).

#### 3.1.3. Baseline to Post RT-Intervention (T1–T3)

Between baseline and the end of RT there was a significant difference between groups for changes in total LBM favouring the supplement group (*p* = 0.05; supplement: ∆ 2.78 ± 1.89 kg; control: ∆ 2.04 ± 2.70 kg; Figure 4). Changes in segmental LBM were not different between groups (arm, *p* = 0.17; leg *p* = 0.27; trunk, *p* = 0.13; see Supplementary Materials, Table S3). There were no between-group differences in fat mass changes (*p* = 0.47; Supplementary Materials, Table S3).

### 3.2. Post Hoc Analysis: Sex Disaggregated Data

#### 3.2.1. Seven-Day Wash-In Phase (T1–T2)

**Figure 5:**
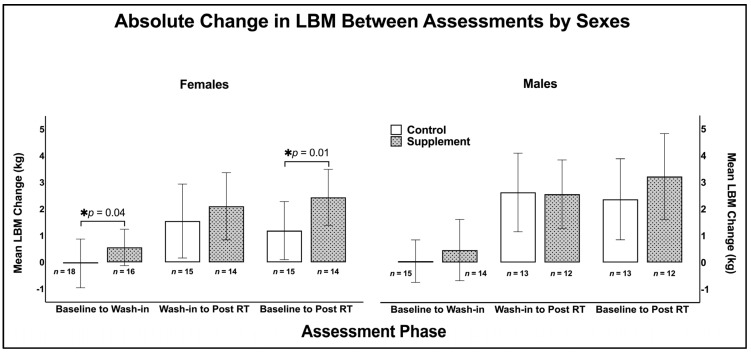
Absolute change (mean ± SD) in lean body mass (kg) by sex. LBM = lean body mass, RT = resistance training; * denotes *p* < 0.05.

With sexes disaggregated, there was a significant difference between groups for changes in total LBM in females favouring the supplement group (*p* = 0.04; supplement: ∆ 0.55 ± 1.11 kg; control: ∆ −0.03 ± 1.06 kg; Figure 5). Change in arm LBM in supplement group females was significantly greater than controls (*p* = 0.03; supplement ∆ 0.54 ± 0.28 kg; control: ∆ 0.37 ± 0.27 kg; Supplementary Materials, Table S3). Change in LBM of the legs was not different between groups for females (*p* = 0.74), but there was a significant difference between groups in trunk LBM favouring the supplement group (*p* = 0.02; supplement: ∆ 0.38 ± 0.88 kg; control: ∆ −0.17 ± 0.83). There were no between-group differences in fat mass changes for females (*p* = 0.75; Supplementary Materials, Table S4).

There were no between-group differences in total (*p* = 0.82), arm (*p* = 0.51), leg (*p* = 0.87), or trunk (*p* = 0.98) LBM change in males (Supplementary Materials, Table S3). There were no between-group differences in fat mass changes for males (*p* = 0.15; Supplementary Materials, Table S4).

#### 3.2.2. Twelve-Week RT Intervention (T2–T3)

There were no differences between groups for total LBM change in females post wash-in to post RT intervention (*p* = 0.10; Figure 5). Change in LBM of the arms was not different between groups in females (*p* = 0.50; Supplementary Materials, Table S3). There was a significant difference between groups in leg LBM change in females favouring the supplement group (*p* = 0.05; supplement: ∆ 1.08 ± 0.74 kg; control: ∆ 0.66 ± 0.72 kg), while there were no between-group differences in trunk LBM changes (*p* = 0.45). There were no group differences in fat mass changes in females (*p* = 0.95; Supplementary Materials, Table S4).

There were no differences between groups for total (*p* = 0.35), arm (*p* = 0.36), leg (*p* = 0.15), and trunk (*p* = 0.62) LBM changes in males. There were no group differences in fat mass changes in males (*p* = 0.59; Supplementary Materials, Table S4).

#### 3.2.3. Baseline–Post RT-Intervention (T1–T3)

Between baseline and the end of RT, there was a significant difference between groups for total LBM change in females favouring the supplement group (*p* = 0.01; supplement: ∆ 2.60 ± 1.64 kg; control: ∆ 1.40 ± 1.59 kg; Figure 5). Change in arm LBM was significantly greater in supplement group females compared to controls (*p* = 0.04; supplement: ∆ 0.54 ± 0.28 kg; control: ∆ 0.37 ± 0.27 kg; Supplementary Materials, Table S3). There were no between-group differences in LBM changes for leg segments in females (*p* = 0.07), whereas the supplement group gained significantly more trunk LBM than controls (*p* = 0.05; supplement: ∆ 0.92 ± 1.05 kg; control: ∆ 0.32 ± 1.01 kg). There were no between-group differences in fat mass changes for females (*p* = 0.96; Supplementary Materials, Table S4).

There were no between-group differences for total LBM change in males (*p* = 0.81). Changes in arm (*p* = 0.16), leg (*p* = 0.91) and trunk (*p* = 0.57) LBM in males was not different between groups. There were no between-group differences in fat mass changes for males (*p* = 0.38; Supplementary Materials, Table S4).

## 4. Discussion

Our results demonstrate that 5 g/day of CrM for seven days led to a significant difference in LBM between groups in the absence of a RT stimulus. This study also found that, when a supplement wash-in period is used prior to commencing RT, 5 g/day of CrM for 12 weeks did not increase LBM to a greater extent than RT alone. The unique design utilised in this study allowed us to distinguish between the isolated effects of CrM and the RT intervention. When the data across the three analyses (T1–T2, T2–T3, T1–T3) are examined, it appears that the driver for the ‘apparent’ additional benefit of CrM is likely coming from changes that occur from supplementation only in the short term.

In the exercise intervention research context, it has not been common practice to have a supplemental wash-in phase prior to baseline assessments. For example, in a recent systematic review demonstrating 1 kg more LBM growth following RT and CrM supplementation, no studies utilised a wash-in [8]. In contrast, previous research revealed that 10 days of CrM, comprising 3 loading and 7 maintenance days, in the absence of a RT stimulus increased LBM by 1 kg [26]. Together with the results of the present study, our findings demonstrate that to prevent over estimation of the effect of CrM combined with RT, it would be beneficial to include a wash-in phase where the research question pertains to the impact of CrM on LBM.

The effects of CrM on body composition have previously been explored predominantly in males, though there may be unique outcomes in females [40]. Our results revealed that the between-group differences in our study were driven by changes in the segmental region of the trunk in the female group only. When data were disaggregated by sex, CrM did not increase LBM in males across any time point. In contrast, in females, CrM increased LBM at the wash-in phase, and when the traditional week 0–13 analysis was utilised. Although there is considerable variability in the LBM outcomes, the significant sex-based differences do suggest a meaningful difference that warrants further discussion. While small shifts occur in extracellular fluid throughout the menstrual cycle, they are too small to impact DXA measurements [41]. However, when CrM is supplemented in the luteal phase of the menstrual cycle, there is a significant increase in total body water, extra cellular fluid, and intracellular fluid when compared to placebo [42]. This could be a potential mechanism of action in the current study. Alternatively, given that females may have higher baseline levels of intramuscular creatine compared to males [43], the increase in creatine consumption in this study could have produced a larger elevation in body water content in females than males. However, we did not track the menstrual cycle or body water content. Given that there are potentially different CrM effects across the lifecycle in females [40], further research is needed to better understand how the effect of supplementation differs between sexes in different scenarios.

Due to its safe and economical use with high precision, the DXA is considered a reference standard to assess changes in LBM following interventions [44,45]. Nonetheless, hydration, stomach contents and food consumption, body position, time of scanning, and prior physical activity could affect whole-body and segmental DXA body composition measurements [46]. In this study, these variables were largely controlled for. However, the type of food consumed before the eight-hour fast and amount of water intake were not standardised and instead were left to the habitual intake of the participant. Although there were no differences in caloric and macronutrients intake between assessments, differences in stomach contents could have influenced DXA measurements [47]. Given the increase in trunk LBM, coupled with the influence of body water and stomach content on DXA measures of LBM, it is plausible that the LBM increases are owing to the changes in fluid in the trunk region.

A limitation of the current study was that we did not assess intramuscular stores of creatine, or creatine uptake levels. However, this is not routinely measured in the RT literature because ~3–5 g/day of CrM supplementation is reportedly sufficient to elicit increases in intramuscular creatine stores [15] and LBM [10], and likely also due to the invasive nature of collecting muscle biopsies. It must be noted that the wash-in phase results fall within the SEM for our DXA machine. Though, that error would not be confined to the CrM group only, and as such, we believe that our data should still be interpreted to reflect a valid between-group difference. Given that we did not track the menstrual cycle or body water content in our study, we were unable to ascertain whether the menstrual cycle impacted fluid retention in the S group. While we did assess changes to caloric and macronutrient intake, no self-reported dietary assessment methods are free of measurement error; as such, the reported values are likely an underestimation of true values [48]. However, under-reporting was likely consistent across the intervention. As such, the lack of reported differences in intake between assessments does likely reflect participants’ adherence to their habitual diets. We did not collect data on long-term dietary patterns, such as animal- or plant-based diets, and given the lower dietary intake of creatine in vegetarians [49], this may have influenced individual responsiveness to supplementation. In addition, while participants received instructions on supplement dosing and their reported compliance was high, ingestion of the supplement was not supervised. Therefore, it is possible that supplementation was, on average, lower than the prescribed 5 g/day. Water consumption was neither measured nor controlled for over the course of the 13-week study. Future research should incorporate measures of hydration when investigating body composition changes following CrM. Finally, while the findings from the present study suggest that more than 5 g/day of CrM may be necessary to augment LBM growth with RT, we only assessed the effects of one dosing strategy. Future research should consider comparing different dosing strategies with a wash-in phase.

## 5. Conclusions

CrM is publicised as the most effective ergogenic nutritional supplement to increase high-intensity exercise capacity and LBM during training. In contrast to previous findings, the results of this study showed that CrM had no additive effect on LBM changes when combined with RT. This is likely due to the increase in LBM following acute CrM supplementation. For long-term CrM supplementation to augment RT, a maintenance dose of greater than 5 g/day may be necessary. Future research should determine the ideal length of a wash-in phase and include measures of hydration status to determine whether any changes to LBM following the wash-in are linked to changes in body water content.

## Figures and Tables

**Table 1:** Baseline characteristics of participants in the control and supplement group (mean ± SD).

	Control	Supplement	Test Statistic	*p* Value
Sample size (*n*, female/male)	33 (18/15)	30 (16/14)		
Age (years)	31 ± 8	30 ± 8	W = 549	0.46
Body mass (kg)	69 ± 14	73 ± 19	W = 432.5	0.39
BMI (kg/m^2^)	23 ± 3	24 ± 5	W = 438	0.44
LBM (kg)	46 ± 10	48 ± 13	t = −0.83 df = 53.05	0.41
Physical activity (mins/week)	52 ± 42	53 ± 32	W = 542	0.50

SD = standard deviation, kg = kilograms, m = meters; mins = minutes.

## Data Availability

The anonymised data collected are available as open data via the Open Science Framework (https://osf.io/4pu2t/).

## Supplementary Materials

The following supporting information can be downloaded at: https://www.mdpi.com/article/10.3390/nu17061081/s1, Table S1: Resistance training program; Table S2: Caloric and Macronutrient Intake in the control and supplement group (mean ± SD); Table S3: Mean absolute change in lean body mass (kg) between assessments; Table S4: Mean absolute change in segmental lean body mass (kg) between; Table S5: Mean absolute change in fat mass (kg) between assessments; Table S6: Mean relative change in lean body mass (% change) between assessments; Table S7: Mean absolute change in body mass (kg) between assessments.

## Abbreviations

The following abbreviations are used in this manuscript:

LBM	lean body mass
RT	resistance training
CrM	creatine monohydrate
DXA	dual energy x-ray absorptiometry
T	time point

## References

[1] Spahillari A., Mukamal K.J., DeFilippi C., Kizer J.R., Gottdiener J.S., Djousse L., Lyles M.F., Bartz T.M., Murthy V.L., Shah R.V. (2016). The association of lean and fat mass with all-cause mortality in older adults: The Cardiovascular Health Study. Nutr. Metab. Cardiovasc. Dis..

[2] Nelson M.E., Fiatarone M.A., Morganti C.M., Trice I., Greenberg R.A., Evans W.J. (1994). Effects of high-intensity strength training on multiple risk factors for osteoporotic fractures. A randomized controlled trial. JAMA.

[3] Szulc P., Beck T.J., Marchand F., Delmas P.D. (2005). Low skeletal muscle mass is associated with poor structural parameters of bone and impaired balance in elderly men–The MINOS study. J. Bone Miner. Res..

[4] Colberg S.R., Sigal R.J., Yardley J.E., Riddell M.C., Dunstan D.W., Dempsey P.C., Horton E.S., Castorino K., Tate D.F. (2016). Physical Activity/Exercise and Diabetes: A Position Statement of the American Diabetes Association. Diabetes Care.

[5] Benito P.J., Cupeiro R., Ramos-Campo D.J., Alcaraz P.E., Rubio-Arias J.A. (2020). A Systematic Review with Meta-Analysis of the Effect of Resistance Training on Whole-Body Muscle Growth in Healthy Adult Males. Int. J. Environ. Res. Public. Health.

[6] Hagstrom A.D., Marshall P.W., Halaki M., Hackett D.A. (2020). The Effect of Resistance Training in Women on Dynamic Strength and Muscular Hypertrophy: A Systematic Review with Meta-analysis. Sports Med..

[7] Kreider R.B., Wilborn C.D., Taylor L., Campbell B., Almada A.L., Collins R., Cooke M., Earnest C.P., Greenwood M., Kalman D.S. (2022). ISSN exercise & sport nutrition review: Research & recommendations. J. Int. Soc. Sports Nutr..

[8] Delpino F.M., Figueiredo L.M., Forbes S.C., Candow D.G., Santos H.O. (2022). Influence of age, sex, and type of exercise on the efficacy of creatine supplementation on lean body mass: A systematic review and meta-analysis of randomized clinical trials. Nutrition.

[9] Desai I., Wewege M.A., Jones M.D., Clifford B.K., Pandit A., Kaakoush N.O., Simar D., Hagstrom A.D. (2024). The Effect of Creatine Supplementation on Resistance Training-Based Changes to Body Composition: A Systematic Review and Meta-analysis. J. Strength. Cond. Res..

[10] Kreider R.B., Kalman D.S., Antonio J., Ziegenfuss T.N., Wildman R., Collins R., Candow D.G., Kleiner S.M., Almada A.L., Lopez H.L. (2017). International Society of Sports Nutrition position stand: Safety and efficacy of creatine supplementation in exercise, sport, and medicine. J. Int. Soc. Sports Nutr..

[11] Leite M.O., Knifis F.W., Machado M. (2023). Creatine Supplementation and Akt/mTOR Pathway: Unraveling the Connection for Optimal Muscle Performance. J. Sports Med. Ther..

[12] Farshidfar F., Pinder M.A., Myrie S.B. (2017). Creatine Supplementation and Skeletal Muscle Metabolism for Building Muscle Mass- Review of the Potential Mechanisms of Action. Curr. Protein Pept. Sci..

[13] Antonio J., Brown A.F., Candow D.G., Chilibeck P.D., Ellery S.J., Forbes S.C., Gualano B., Jagim A.R., Kerksick C., Kreider R.B. (2025). Part II. Common questions and misconceptions about creatine supplementation: What does the scientific evidence really show?. J. Int. Soc. Sports Nutr..

[14] Gualano B., Macedo A.R., Alves C.R., Roschel H., Benatti F.B., Takayama L., de Sa Pinto A.L., Lima F.R., Pereira R.M. (2014). Creatine supplementation and resistance training in vulnerable older women: A randomized double-blind placebo-controlled clinical trial. Exp. Gerontol..

[15] Hultman E., Soderlund K., Timmons J.A., Cederblad G., Greenhaff P.L. (1996). Muscle creatine loading in men. J. Appl. Physiol..

[16] Willoughby D.S., Rosene J.M. (2001). Effects of oral creatine and resistance training on myosin heavy chain expression. Med. Sci. Sports Exerc..

[17] Willoughby D.S., Rosene J.M. (2003). Effects of oral creatine and resistance training on myogenic regulatory factor expression. Med. Sci. Sports Exerc..

[18] Cooper R., Naclerio F., Allgrove J., Jimenez A. (2012). Creatine supplementation with specific view to exercise/sports performance: An update. J. Int. Soc. Sports Nutr..

[19] Wyss M., Kaddurah-Daouk R. (2000). Creatine and creatinine metabolism. Physiol. Rev..

[20] Andre T.L., Gann J.J., McKinley-Barnard S.K., Willoughby D.S. (2016). Effects of Five Weeks of Resistance Training and Relatively-Dosed Creatine Monohydrate Supplementation on Body Composition and Muscle Strength, and Whole-Body Creatine Metabolism in Resistance-Trained Males. Int. J. Kinesiol. Sports Sci..

[21] Jagim A.R., Oliver J.M., Sanchez A., Galvan E., Fluckey J., Riechman S., Greenwood M., Kelly K., Meininger C., Rasmussen C. (2012). A buffered form of creatine does not promote greater changes in muscle creatine content, body composition, or training adaptations than creatine monohydrate. J. Int. Soc. Sports Nutr..

[22] Rawson E.S., Stec M.J., Frederickson S.J., Miles M.P. (2011). Low-dose creatine supplementation enhances fatigue resistance in the absence of weight gain. Nutrition.

[23] Spillane M., Schoch R., Cooke M., Harvey T., Greenwood M., Kreider R., Willoughby D.S. (2009). The effects of creatine ethyl ester supplementation combined with heavy resistance training on body composition, muscle performance, and serum and muscle creatine levels. J. Int. Soc. Sports Nutr..

[24] Powers M.E., Arnold B.L., Weltman A.L., Perrin D.H., Mistry D., Kahler D.M., Kraemer W., Volek J. (2003). Creatine supplementation increases total body water without altering fluid distribution. J. Athl. Train..

[25] Ribeiro A.S., Avelar A., Kassiano W., Nunes J.P., Schoenfeld B.J., Aguiar A.F., Trindade M.C.C., Silva A.M., Sardinha L.B., Cyrino E.S. (2020). Creatine Supplementation Does Not Influence the Ratio Between Intracellular Water and Skeletal Muscle Mass in Resistance-Trained Men. Int. J. Sport Nutr. Exerc. Metab..

[26] Safdar A., Yardley N.J., Snow R., Melov S., Tarnopolsky M.A. (2008). Global and targeted gene expression and protein content in skeletal muscle of young men following short-term creatine monohydrate supplementation. Physiol. Genom..

[27] Dallal G.E. Randomization.com. http://www.randomization.com.

[28] Harris P.A., Taylor R., Minor B.L., Elliott V., Fernandez M., O’Neal L., McLeod L., Delacqua G., Delacqua F., Kirby J. (2019). The REDCap consortium: Building an international community of software platform partners. J. Biomed. Inform..

[29] Harris P.A., Taylor R., Thielke R., Payne J., Gonzalez N., Conde J.G. (2009). Research electronic data capture (REDCap)--a metadata-driven methodology and workflow process for providing translational research informatics support. J. Biomed. Inform..

[30] Norton K. (2012). New Australian standard for adult pre-exercise screening. Sport Health.

[31] Lee P.H., Macfarlane D.J., Lam T.H., Stewart S.M. (2011). Validity of the International Physical Activity Questionnaire Short Form (IPAQ-SF): A systematic review. Int. J. Behav. Nutr. Phys. Act..

[32] Schoenfeld B.J., Ogborn D., Krieger J.W. (2017). Dose-response relationship between weekly resistance training volume and increases in muscle mass: A systematic review and meta-analysis. J. Sports Sci..

[33] Helms E.R., Cronin J., Storey A., Zourdos M.C. (2016). Application of theRepetitions in Reserve-Based Rating ofPerceived Exertion Scalefor Resistance Training. Strength Cond. J..

[34] Morton R.W., Colenso-Semple L., Phillips S.M. (2019). Training for strength and hypertrophy: An evidence-based approach. Curr. Opin. Physiol..

[35] Cohen J., Cohen P., West S.G., Aiken L.S. (2013). Applied Multiple Regression/Correlation Analysis for the Behavioral Sciences.

[36] Allen J.M., Mailing L.J., Niemiro G.M., Moore R., Cook M.D., White B.A., Holscher H.D., Woods J.A. (2018). Exercise Alters Gut Microbiota Composition and Function in Lean and Obese Humans. Med. Sci. Sports Exerc..

[37] Bycura D., Santos A.C., Shiffer A., Kyman S., Winfree K., Sutliffe J., Pearson T., Sonderegger D., Cope E., Caporaso J.G. (2021). Impact of Different Exercise Modalities on the Human Gut Microbiome. Sports.

[38] Cronin O., Barton W., Skuse P., Penney N.C., Garcia-Perez I., Murphy E.F., Woods T., Nugent H., Fanning A., Melgar S. (2018). A Prospective Metagenomic and Metabolomic Analysis of the Impact of Exercise and/or Whey Protein Supplementation on the Gut Microbiome of Sedentary Adults. mSystems.

[39] R Studio Team (2020). RStudio: Integrated Development for R.

[40] Smith-Ryan A.E., Cabre H.E., Eckerson J.M., Candow D.G. (2021). Creatine Supplementation in Women’s Health: A Lifespan Perspective. Nutrients.

[41] Hicks C.S., McLester C.N., Esmat T.A., McLester J.R. (2017). A Comparison of Body Composition Across Two Phases of the Menstrual Cycle Utilizing Dual-Energy X-Ray Absorptiometry, Air Displacement Plethysmography, and Bioelectrical Impedance Analysis. Int. J. Exerc. Sci..

[42] Moore S.R., Gordon A.N., Cabre H.E., Hackney A.C., Smith-Ryan A.E. (2023). A Randomized Controlled Trial of Changes in Fluid Distribution across Menstrual Phases with Creatine Supplementation. Nutrients.

[43] Forsberg A.M., Nilsson E., Werneman J., Bergstrom J., Hultman E. (1991). Muscle composition in relation to age and sex. Clin. Sci..

[44] Buckinx F., Landi F., Cesari M., Fielding R.A., Visser M., Engelke K., Maggi S., Dennison E., Al-Daghri N.M., Allepaerts S. (2018). Pitfalls in the measurement of muscle mass: A need for a reference standard. J. Cachexia Sarcopenia Muscle.

[45] Erlandson M.C., Lorbergs A.L., Mathur S., Cheung A.M. (2016). Muscle analysis using pQCT, DXA and MRI. Eur. J. Radiol..

[46] Hangartner T.N., Warner S., Braillon P., Jankowski L., Shepherd J. (2013). The Official Positions of the International Society for Clinical Densitometry: Acquisition of dual-energy X-ray absorptiometry body composition and considerations regarding analysis and repeatability of measures. J. Clin. Densitom..

[47] Shiel F., Persson C., Furness J., Simas V., Pope R., Climstein M., Hing W., Schram B. (2018). Dual energy X-ray absorptiometry positioning protocols in assessing body composition: A systematic review of the literature. J. Sci. Med. Sport.

[48] Cade J.E., Warthon-Medina M., Albar S., Alwan N.A., Ness A., Roe M., Wark P.A., Greathead K., Burley V.J., Finglas P. (2017). DIET@NET: Best Practice Guidelines for dietary assessment in health research. BMC Med..

[49] Burke D.G., Chilibeck P.D., Parise G., Candow D.G., Mahoney D., Tarnopolsky M. (2003). Effect of creatine and weight training on muscle creatine and performance in vegetarians. Med. Sci. Sports Exerc..

