# Supplementary material for: The Effect of Creatine Supplementation on Lean Body Mass with and Without Resistance Training
Source: Nutrients. 2025 Mar 19;17(6):1081. doi: 10.3390/nu17061081 (PMC11944689; doi:10.3390/nu17061081)
Supplement: Supplementary file 1 [file nutrients-17-01081-s001.zip › nutrients-3529713-supplementary.pdf]

The effect of creatine supplementation on lean body mass with and without resistance training

Supplementary Material

Table of Contents

Table S1: Resistance training program. **3**

Table S2: Caloric and Macronutrient Intake in the control and supplement group (mean ± SD)

Table S3: Mean absolute change in lean body mass (kg) between assessments. **5**

Table S4: Mean absolute change in segmental lean body mass (kg) between assessments. **6**

Table S5: Mean absolute change in fat mass (kg) between assessments. **7**

Table S6: Mean relative change in lean body mass (% change) between assessments. **7**

Table S7: Mean absolute change in body mass (kg) between assessments. **8**

Table S1. Resistance Training Program

WEEKS 1 – 4			
**DAY**	**EXERCISE**	**SETS**	**REP MAX**
1	Horizontal leg press	4	8
	Seated row, V-bar	4	8
	Chest press machine, natural grip	4	8
	Leg curl machine	4	8
	Cable rope triceps press down	3	8
		1	To failure
2	DB Split squat	4	6
	Lat pulldown, supinated	4	6
	Standing landmine press (2 hands)	4	6
	Landmine Romanian DL (bilateral)	4	6
	Cable straight bar bicep curl	3	6
		1	To failure
3	Leg extension	4	10
	Seated row, wide	4	10
	Chest press machine, narrow	4	10
	45-degree hip extension	4	10
	Cable straight bar triceps pressdown	3	10
		1	To failure
**WEEKS 5 – 8**			
**DAY**	**EXERCISE**	**SETS**	**REP MAX**
1	FFE split squat	4	8
	Lat pull down, pronated	3	8
		1	To failure
	Single arm landmine press, standing	4	8
	DB Romanian deadlift	4	8
	Cable V-bar triceps extension	3	8
		1	To failure
2	Dual DB cyclist squat	4	10
	Seated row, rope	3	10
		1	To failure
	BB floor press	4	10
	45-degree hip extension	4	10
	Seated DB bicep curl	3	10
		1	To failure
3	Incline leg press	4	12
	Kneeling DB row	3	12
		1	To failure
	DB bench press, narrow	4	12
	2 up, 1 down leg curl	4	12
	Cable bicep curl, rope	3	12
		1	To failure

WEEKS 9 – 12			
**DAY**	**EXERCISE**	**SETS**	**REP MAX**
1	BB Split squat	4	8
	3-point DB row	3	8
		1	To failure
	Incline DB bench press	4	8
		1	To failure
	BB Romanian deadlift	4	8
	Cable overhead triceps extension	3	8
		1	To failure
2	Horizontal squat machine	4	10
	DB chainsaw row	3	10
		1	To failure
	DB Z-press	3	10
		1	To failure
	Leg curl	4	10
	BB bicep curl	3	10
		1	To failure
3	Incline leg press	4	12
	V-bar lat pull down	3	12
		1	To failure
	Chest press machine, natural	3	12
		1	To failure
	DB Split stance Romanian deadlift	4	12
	DB skull crushers	3	12
		1	To failure

Rep max = repetition maximum (RM); DB = dumbbell; BB = barbell

Table S2. Caloric and Macronutrient Intake in the control and supplement group (mean ± SD)

	T1			T2			T3		
	**Control**	**Supplement**	***p* values**	**Control**	**Supplement**	***p* values**	**Control**	**Supplement**	***p* values**
Total Calories (kcal/day)	1644 ± 444	1641 ± 493	0.98	1506 ± 408	1626 ± 553	0.47	1601 ± 480	1658 ± 529	0.76
Carbohydrates (g/day)	213 ± 154	166 ± 56	0.04*	167 ± 66	166 ± 54	0.16	173 ± 67	171 ± 64	0.21
Fat (g/day)	65 ± 19	65 ± 24	0.92	57 ± 18	65 ± 27	0.27	66 ± 22	62 ± 25	0.70
Protein (g/day)	74 ± 24	75 ± 40	0.92	65 ± 19	78 ± 38	0.25	75 ± 28	87 ± 36	0.42

SD = standard deviation, kcal = kilocalories, g = grams

Table S3. Absolute change in lean body mass (kg) between assessments (mean ± SD)

	**Wash-in**			**Post Wash-in to Post RT**			**Baseline to Post RT**		
	**Sexes Combined**	**Female**	**Male**	**Sexes Combined**	**Female**	**Male**	**Sexes Combined**	**Female**	**Male**
**Control**	0.00 ± 1.20	-0.03 ± 1.06	0.21 ± 1.25	2.11 ± 1.71	1.47 ± 1.51	2.88 ± 1.87	2.04 ± 1.82	1.40 ± 1.59	2.97 ± 2.06
**Supp.**	**0.51 ± 1.26***	**0.55 ± 1.11***	0.30 ± 1.30	2.24 ± 1.79	2.20 ± 1.57	2.28 ± 1.96	**2.78 ± 1.89***	**2.60 ± 1.64***	2.80 ± 2.16

* Significantly different change between groups, (p ≤ 0.05) using a 95% confidence interval

Table S4 Absolute change in segmental lean body mass (kg) between assessments (mean ± SD)

	Wash-in			Post Wash-in to Post RT			Baseline to Post RT		
	**Sexes Combined**	**Female**	**Male**	**Sexes Combined**	**Female**	**Male**	**Sexes Combined**	**Female**	**Male**
**Arms**									
**Control**	0.05 ± 0.31	0.02 ± 0.14	0.08 ± 0.45	0.58 ± 0.44	0.38 ± 0.25	0.74 ± 0.61	0.61 ± 0.59	0.37 ± 0.27	0.79 ± 0.82
**Supp.**	0.12 ± 0.33	**0.10 ± 0.15***	0.17 ± 0.49	0.63 ± 0.45	0.43 ± 0.26	0.94 ± 0.68	0.78 ± 0.61	**0.54 ± 0.28***	1.19 ± 0.90
**Legs**									
**Control**	0.05 ± 0.54	0.10 ± 0.36	0.00 ± 0.71	0.82 ± 0.91	0.66 ± 0.72	1.11 ± 0.93	0.82 ± 1.00	0.75 ± 0.68	0.95 ± 1.31
**Supp.**	0.08 ± 0.57	0.07 ± 0.38	0.03 ± 0.76	0.93 ± 0.94	**1.08 ± 0.74***	0.628 ± 1.03	1.05 ± 1.04	1.11 ± 0.71	0.90 ± 1.44
**Trunk**									
**Control**	-0.10 ± 0.88	-0.17 ± 0.83	0.10 ± 0.90	0.74 ± 1.16	0.49 ± 0.91	0.96 ± 1.43	0.62 ± 1.92	0.32 ± 1.01	1.09 ± 1.39
**Supp.**	**0.32 ± 0.92***	**0.38 ± 0.88***	0.10 ± 0.97	0.72 ± 1.20	0.69 ± 0.94	0.72 ± 1.57	0.99 ± 1.24	**0.92 ± 1.05***	0.81 ± 1.53

Supp. = Supplement group; * Significantly different change between groups, ($p \leq 0.05$) using a 95% confidence interval

Table S5. Absolute change in fat mass (kg) between assessments (mean ± SD)

	**Wash-in**			**Post Wash-in to Post RT**			**Baseline to Post RT**		
	**Sexes Combined**	**Female**	**Male**	**Sexes Combined**	**Female**	**Male**	**Sexes Combined**	**Female**	**Male**
**Control**	-0.01 ± 0.54	0.01 ± 0.50	-0.02 ± 0.50	-0.39 ± 2.17	-0.25 ± 1.76	-0.54 ± 2.49	-0.41 ± 2.34	-0.29 ± 1.89	-0.55 ± 2.66
**Supp.**	-0.12 ± 0.56	-0.03 ± 0.53	-0.23 ± 0.52	-0.59 ± 2.25	-0.27 ± 1.83	-0.96 ± 2.59	-0.76 ± 2.44	-0.32 ± 1.96	-1.28 ± 2.76

Supp. = Supplement group

Table S6. Relative change in lean body mass (% change) between assessments (mean ± SD)

	**Wash-in**			**Post Wash-in to Post RT**			**Baseline to Post RT**		
	**Sexes Combined**	**Female**	**Male**	**Sexes Combined**	**Female**	**Male**	**Sexes Combined**	**Female**	**Male**
**Control**	0.05 ± 0.35	0.06 ± 2.88	0.37 ± 2.19	4.32 ± 3.66	3.73 ± 3.72	5.07 ± 3.36	4.23 ± 4.0	3.69 ± 4.0	5.26 ± 3.85
**Supp.**	**1.12 ± 8.68***	1.49 ± 2.95	0.34 ± 2.27	4.78 ± 3.81	5.49 ± 3.85	3.86 ± 3.52	**5.91 ± 4.15***	**6.64 ± 4.13***	4.62 ± 4.03

Supp. = Supplement group; * Significantly different change between groups, ($p \leq 0.05$) using a 95% confidence interval

Table S7. Absolute change in body mass (kg) between assessments (mean ± SD)

	Wash-in			Post Wash-in to Post RT			Baseline to Post RT		
	Sexes Combined	Female	Male	Sexes Combined	Female	Male	Sexes Combined	Female	Male
**Control**	0.00 ± 0.76	-0.03 ± 0.84	0.04 ± 0.69	1.70 ± 1.40	1.30 ± 1.07	2.10 ± 1.61	1.65 ± 1.32	1.23 ± 0.90	2.07 ± 1.56
**Supp.**	0.40 ± 0.94	0.54 ± 0.80	0.25 ± 1.09	1.23 ± 2.09	1.40 ± 2.24	1.08 ± 2.05	1.59 ± 2.00	2.01 ± 2.26	1.21 ± 1.77

Supp. = Supplement group
